# Single-cell RNA sequencing revealed potential targets for immunotherapy studies in hepatocellular carcinoma

**DOI:** 10.1038/s41598-023-46132-w

**Published:** 2023-11-01

**Authors:** Zhouhua Xie, Jinping Huang, Yanjun Li, Qingdong Zhu, Xianzhen Huang, Jieling Chen, Cailing Wei, Shunda Luo, Shixiong Yang, Jiamin Gao

**Affiliations:** 1 Laboratory of Infectious Disease, HIV/AIDS Clinical Treatment Center of Guangxi (Nanning) and The Fourth People’s Hospital of Nanning, Nanning, 530023 Guangxi Zhuang Autonomous Region China; 2 Department of Tuberculosis, HIV/AIDS Clinical Treatment Center of Guangxi (Nanning) and The Fourth People’s Hospital of Nanning, Nanning, 530023 China; 3 Department of Infectious Diseases, HIV/AIDS Clinical Treatment Center of Guangxi (Nanning) and The Fourth People’s Hospital of Nanning, Nanning, 530023 China; 4 Department of Clinical Laboratory, HIV/AIDS Clinical Treatment Center of Guangxi (Nanning) and The Fourth People’s Hospital of Nanning, Nanning, 530023 China; 5 Administrative Office, HIV/AIDS Clinical Treatment Center of Guangxi (Nanning) and The Fourth People’s Hospital of Nanning, Nanning, 530023 China

**Keywords:** Tumour heterogeneity, Immunotherapy

## Abstract

Hepatocellular carcinoma (HCC) is a solid tumor prone to chemotherapy resistance, and combined immunotherapy is expected to bring a breakthrough in HCC treatment. However, the tumor and tumor microenvironment (TME) of HCC is highly complex and heterogeneous, and there are still many unknowns regarding tumor cell stemness and metabolic reprogramming in HCC. In this study, we combined single-cell RNA sequencing data from 27 HCC tumor tissues and 4 adjacent non-tumor tissues, and bulk RNA sequencing data from 374 of the Cancer Genome Atlas (TCGA)-liver hepatocellular carcinoma (LIHC) samples to construct a global single-cell landscape atlas of HCC. We analyzed the enrichment of signaling pathways of different cells in HCC, and identified the developmental trajectories of cell subpopulations in the TME using pseudotime analysis. Subsequently, we performed transcription factors regulating different subpopulations and gene regulatory network analysis, respectively. In addition, we estimated the stemness index of tumor cells and analyzed the intercellular communication between tumors and key TME cell clusters. We identified novel HCC cell clusters that specifically express HP (HCC_HP), which may lead to higher tumor differentiation and tumor heterogeneity. In addition, we found that the HP gene expression-positive neutrophil cluster (Neu_AIF1) had extensive and strong intercellular communication with HCC cells, tumor endothelial cells (TEC) and cancer-associated fibroblasts (CAF), suggesting that clearance of this new cluster may inhibit HCC progression. Furthermore, ErbB signaling pathway and GnRH signaling pathway were found to be upregulated in almost all HCC tumor-associated stromal cells and immune cells, except NKT cells. Moreover, the high intercellular communication between HCC and HSPA1-positive TME cells suggests that the immune microenvironment may be reprogrammed. In summary, our present study depicted the single-cell landscape heterogeneity of human HCC, identified new cell clusters in tumor cells and neutrophils with potential implications for immunotherapy research, discovered complex intercellular communication between tumor cells and TME cells.

## Introduction

Hepatocellular carcinoma (HCC) is becoming one of the most common and deadly malignancies worldwide. Treatment options for HCC include tumor resection, liver transplantation, or liver ablation, but they only apply to part of the patients with an early diagnosis, with approximately 50% of HCC patients undergoing systemic therapy^1^. HCC has traditionally been considered as a chemoresistant solid tumor, but the advent of immunotherapy has brought a new prospects for cancer therapy^2,3^. However, the efficacy of immunotherapy for HCC is significantly influenced by the heterogeneity of the tumor's microenvironment (TME)^4^. The TME is an ecosystem created by cancer cells composed of components from both tumor and hosts. The high heterogeneity of tumors and TME in HCC results in dramatic differences in immunotherapy sensitivity between patients and it remains unclear how different TME subtypes are clinically relevant to HCC^5^.

As it is known, cancer cells adjust their metabolism to maintain high proliferation and survive in adverse environments with low oxygen and nutrient deficiency, thus metabolic reprogramming is most common in the TME^6^. The dynamic interactions between cancer cells and the TME cells are critical in generating heterogeneity, clonal evolution, and enhancing multi-drug resistance in tumor cells^7^. Single-cell RNA-sequencing (scRNA-seq) allows us to study the various components of the TME and their interactions at a higher resolution^8^. Although there are many single-cell studies on HCC, most of these studies usually focus on specific cell types of interest. The global single-cell landscape heterogeneity of HCC remains largely unknown.

Cancer cells have phenotypic plasticity that not only promotes cellular diversity and tumor evolution, but also reprograms and/or transdifferentiates to cancer stem cell-like cells (CSC)^9^. Therefoer, assessment of stemness in HCC malignant cells can help us to understand tumor development and differentiation, so as to inhibit it. To date, the major focus of cancer immunotherapy has been the interruption of immune checkpoints that suppress anti-tumour lymphocytes. In addition to lymphocytes, the HCC environment includes many other immune cell types, of which neutrophils are emerging as important contributors to the pathogenesis of HCC pathogenesis^10,11^. Simultaneous targeting of tumor cells and surrounding growth-supporting immune cells is a promising strategy to modify the TME and enhance host antitumor immune responses^12^.

Thus, to identify the potential transcriptomic changes of clinical significance in HCC, we combined scRNA-seq data from the Gene Expression Omnibus (GEO) database, which consisted of 27 HCC tumor tissues and 4 adjacent non-tumor tissues, with bulk RNA sequencing data from 374 samples of the Cancer Genome Atlas (TCGA)-liver hepatocellular carcinoma (LIHC) samples to construct a global single-cell landscape atlas for HCC. We conducted an analysis of the dynamic changes observed in specific subgroups of cells within the tumor immune microenvironment, as well as the corresponding signaling pathways that exhibited significant correlations. In addition, we revealed the developmental trajectory of various cell subgroups and identified their key transcriptional regulatory targets. For HCC cells, we computed copy number variations (CNVs) and assessed cancer stemness scores.

Our study investigated the heterogeneity of single cells in human HCC. We discovered novel cell clusters in both tumor cells (HCC_HP) and neutrophils (Neu_AIF1) through our research. These findings hold significant implications for the field of immunotherapy research. Furthermore, our study unveiled intricate intercellular communication between tumor cells and cells in the TME. Additionally, we formulated a prognostic model with high confidence to assess potential risks for distinct cell subclusters. Based on our results, we postulated an exosome-mediated metabolic reprogramming process in HCC. Significant up-regulation of the ErbB signaling pathway, along with multicellular communication involving HSPA1-positive cells, supports this hypothesis. Identifying this metabolic reprogramming process offers valuable insights for future immunotherapy studies.

## Method

### Single-cell RNA sequencing data sources and preprocessing

HCC-related scRNA-seq data were obtained from the Gene Expression Omnibus (GEO) database: GSE125449 (GPL18573)^13^, GSE210679 (GPL20795)^14^, and GSE189903 (GPL24676)^15^, containing a total of 27 HCC tumor tissues and 4 adjacent non-tumor tissues (Supplementary Table 1). Single-cell data was integrated used the IntegrateData function of the Seurat package. We performed the quality control (QC) of the integrated single-cell data, filtering out cells with the highest and lowest number of 1% and cells with more than 10% of mitochondrial genes expression. Cells were clustered according to default parameters and visualized for dimension reduction by the Uniform Manifold Approximation and Projection (UMAP) method^16–18^. The definition of cell type was defined based on the known marker genes (Supplementary Table 2).

### Bulk RNA-sequencing data sources and preprocessing

TCGA-LIHC data were obtained from TCGA database containing bulk RNA-seq data and corresponding clinical information for 374 HCC samples (https://portal.gdc.cancer.gov/projects/TCGA-LIHC). In the HCC cohort, the bulk RNA-seq gene expression data were used log2 transform to analyze differentially expressed genes (DEGs) between high and low immune infiltration groups^19^.

### Differential expression gene (DEG) analysis

DEGs of different clusters in the HCC and adjacent non-tumor tissues were using the FindMarkers function in the Seurat package. Additionally, DEGs between normal liver tissues and tumor tissues were screened by limma package in TCGA-LIHC data. DEGs with adjusted P < 0.05 and | log fold change (log FC) |> 0.5 were considered significant^20^.

### Estimation of single cells CNV in HCC

Interindividual CNVs are used to determine the genomic contribution of complex traits in HCC^21–23^. In our study, the R package InferCNV (v1.6.0) was used to calculate CNVs in cells derived from HCC tumor tissue, applying default parameters (https://github.com/broadinstitute/inferCNV).

### Enrichment analysis of KEGG

To explore the potential biological functions involved in HCC microenvironment cells, enrichment analysis of Kyoto Encyclopedia of Genes and Genomes pathways^24–26^ was performed on top 50 clustered marker genes. KEGG analyses were performed using the clusterProfiler package^27^, biological processes and pathways with *p* < 0.05 was considered significant in statistically.

### Cellular communication analysis

Cellular communication processes and their regulation play an important role in cancer progression. In this study, we performed a cell–cell communication analysis using the iTALK package (https://github.com/Coolgenome/iTALK), to depict the receptor-ligand interactions pairs between different cells in the HCC microenvironment. Considering the testing efficiency and computational burden, we focused on the tumor cells of interest and TME cell populations, and only 350–550 cells in each cell population were randomly selected for analysis.

### Transcription factors (TFs)-based gene regulatory network

Gene regulatory networks (GRNs) is the mechanism controlling gene expression in the organism, ensure coordinated cell behavior and fate decisions^28^. To construct GRNs and identify transcription factors (TFs) that regulate each gene module, we performed GRN analysis using the R package SCENIC (v1.1.3) based on default parameters^29,30^. We obtained transcription factor binding profiles from the JASPAR database (https://jaspar.genereg.net)^31^, construct the patterns of TFs regulating each co-expressed gene module.

### Pseudotime analysis

To infer the developmental trajectories of each cell type, we performed a pseudotime analysis using the R package Monocle 3 (https://cole-trapnell-lab.github.io/monocle3), and the results were dimensional clustering and visualization by the UMAP method.

### Construction of the prognostic model

We used the top 30 genes expressed by the cell cluster specifically to characterize the relative abundance of the cells, and then used multivariate COX analysis to analyze the relationship between cells relative abundance and clinical features. Using the default cut-off value, the samples were divided into high- and low- abundance groups. Overall survival (OS) and relapse-free survival (RFS) were determined using R package survival and survminer, and the potential risk score for cell relative abundance was calculated using the formula proposed by Li et al.^23^.

### Stemness score of tumor cells

Cancer stem cells are the main force of tumor initiation, self-proliferation, and renewal, a small amount of cancer stem cells is sufficient to initiate new tumor formation, causing recurrence and metastasis^32^. We evaluated the cell stemness score based on R package TCGAbiolinks, cell stemness score calculated by the stemness index for the expression profile of each sample with default parameters^33,34^.

### Estimate systems immune response

Utilizing RNA-seq data, EaSIeR provides a comprehensive description of the immune response in complex and dynamic systems, such as tumors. We used the R package easier^35^ for predicting immune response in HCC samples. We classified the samples based on the median of the scoring results. Those samples that scored above the median were grouped into the immunotherapy response group, while the samples that scored below were categorized as the immunotherapy resistance group. This allowed us to obtain the subtypes of HCC cells between HCC patients with immunotherapy response and HCC patients with immunotherapy resistance.

### Statistical analysis

Statistical analyses of our study were performed using R (https://www.rproject.org/). The gene expression levels were analyzed by an unpaired t test, when *p* < 0.05 were considered significant.The Bioinforcloud platform is the main platform used for data analysis in our study (http://www.bioinforcloud.org.cn).

## Result

### Single cellular landscape of tumor microenvironment in HCC

**Figure 1:**
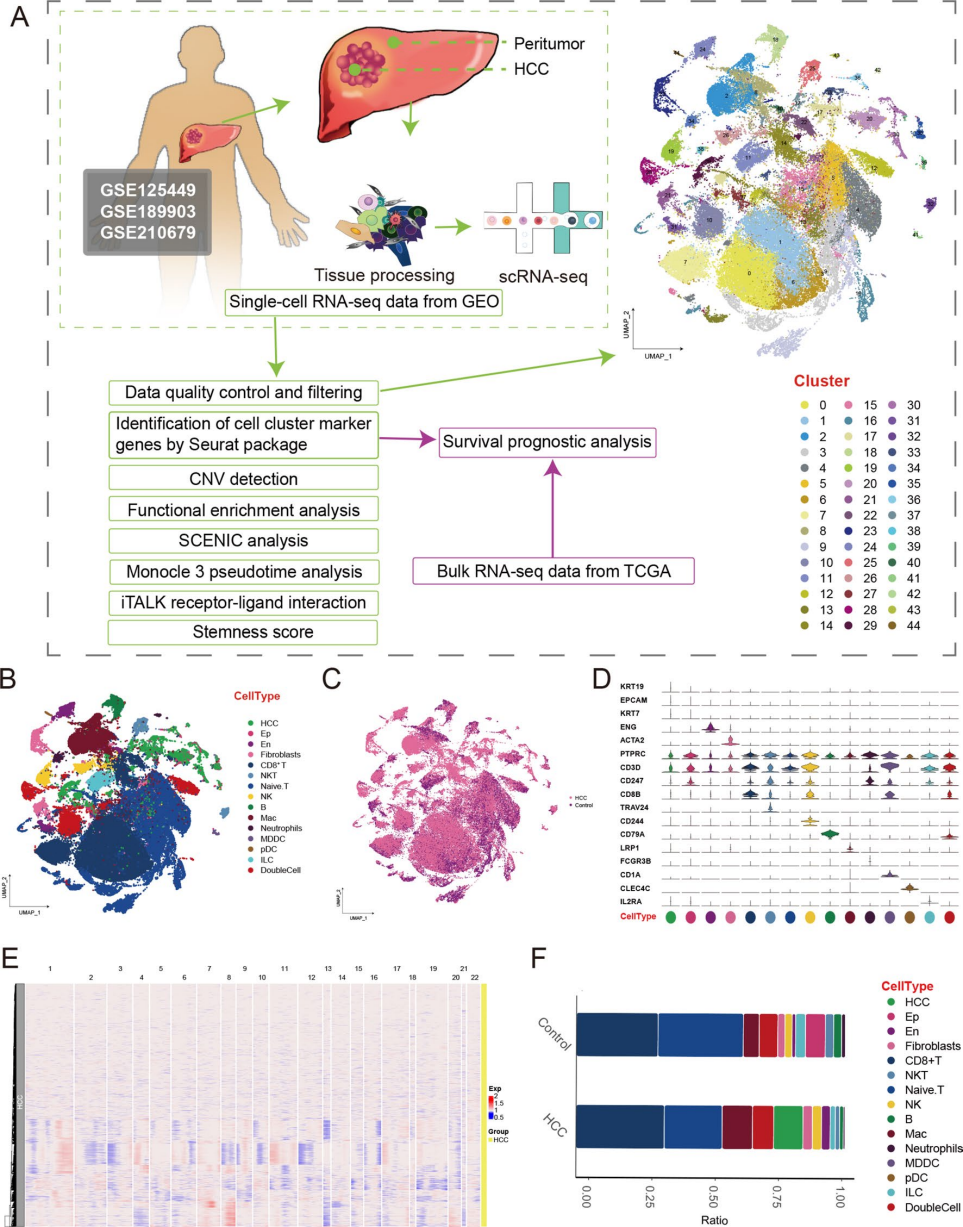
Single cellular landscape of tumor microenvironment in HCC. (**A**) Schematic diagram of our workflow. HCC tumor and adjacent tissues were processing and dissociated into single cells, and sequenced using 10 × Genomics platform. 45 cell clusters were identified of 89,655 cells. b. Expression correlation between single-cell clusters. (**B**) The cell types of single cells mapping HCC includes tumor cells (HCC), epithelial cells (Ep), endothelial cells (En), fibroblasts (Fib), CD8 + T cells, NKT cells, Naive.T cells, NK cells, B cells, macrophages (Mac), neutrophils (Neu), monocyte-derived dendritic cells (MDDC), plasmacytoid dendritic cell (pDC), and Innate lymphoid cells (ILC). (**C**) Single-cell atlas reveals the cell distribution in HCC patients and the control group. (**D**) Violin plots showing marker genes for 15 distinct cell types. (**E**) The heatmap shows the CNV levels of HCC in chromosomes 1 to 22. The rows represent the HCC samples, while the columns correspond to the specific chromosome numbers. (**F**) Bar plots showing the proportion of cell types in each sample. HCC, Hepatocellular carcinoma; scRNA-seq, single-cell RNA-sequencing; UMAP, Uniform Manifold Approximation and Projection.

As shown in Fig. 1A, we constructed a global single-cell landscape of human HCC. After QC, we divided 89,655 cells into 45 cell clusters, which were identified into 15 distinct cell types based on known markers (Fig. 1B), including HCC cells, epithelial cells (Ep), endothelial cells (En), fibroblasts (Fib), CD8^+^ T cells, NKT cells, Naive.T cells, NK cells, B cells, macrophages (Mac), neutrophils (Neu), monocyte-derived dendritic cells (MDDC), plasmacytoid dendritic cell (pDC), and innate lymphoid cells (ILC). Furthermore, there were some cells that detected expression of both T cell markers (CD3, CD247) and B cell markers (CD79A, CD79B) that were defined as DoubleCell. Furthermore, we have showed the single-cell atlas of tumor samples in comparison with normal controls (Fig. 1C). Each cell marker exhibited a specific gene expression for the cell cluster, confirmed the accuracy of cell identity (Fig. 1D; Supplementary Table 3). Chromosomal CNV analysis based on single-cell expression profiles revealed multiple copy events in chromosomes 1 to 22 in HCC samples (Fig. 1E). The proportion of En and Mac cells was significantly increased in HCC compared to control (Fig. 1F). In conclusion, we constructed a global single-cell atlas of human HCC with precise cell type annotation, which is important for our in-depth analysis of the heterogeneity of the HCC tumor and tumor microenvironment.

### Revealed malignant cell subtypes that important for HCC heterogeneity

**Figure 2:**
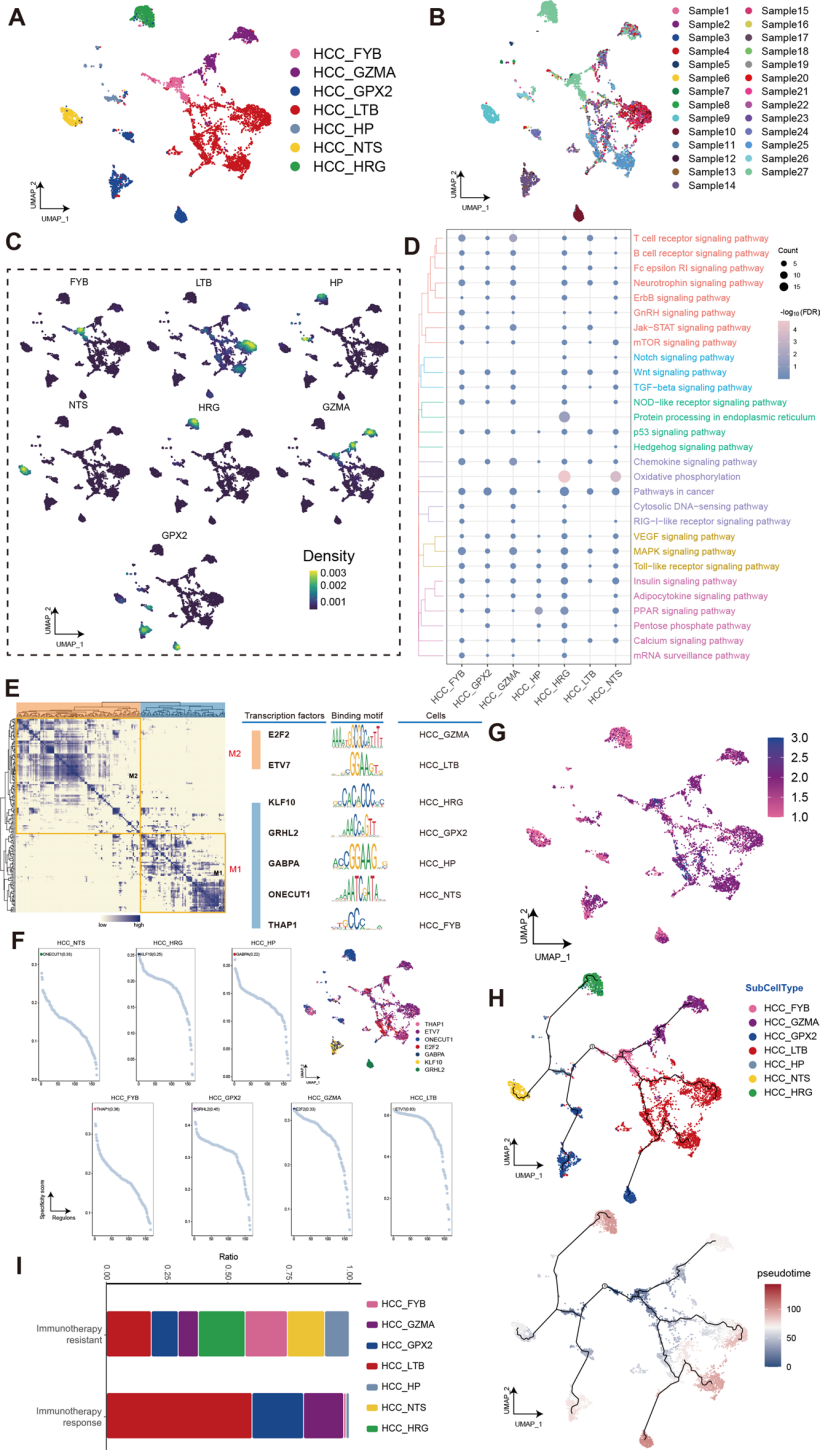
Identification of the tumor cells clusters in human HCC. (**A**) Single-cell atlas shows the malignant cellular clusters of HCC. (**B**) Single-cell atlas shows cell clusters of tumor cells in related samples. (**C**) Marker genes for the distinct tumor cells clusters. (**D**) Biological pathways in distinct clusters of HCC. Bubble colors indicate the significance (−log_10_(FDR)) of enrichment, whereas bubble sizes correspond to the number of genes (Count) enriched in the pathway. (**E**) Transcription factors of tumor cells clusters in a co-expression pattern. Left: Heat map identified co-expression modules; Middle: major transcription factors and their binding sequences; Right: cell clusters of transcription factors. (**F**) The single-cell atlas showcases the transcription factors regulating gene expression in specific tumor cells clusters. The scatterplot of each tumor cells cluster highlights the top-ranked regulon with the highest score. (**G**) Cell stemness is scored for different cell clusters of HCC. (**H**) Single-cell atlas map the trajectory and pseudotime values of HCC malignent cells progression. (**I**) Differences in the abundance of tumor cells clusters between immunotherapy response and immunotherapy-resistant HCC patients.

Seven malignant cell clusters (HCC_FYB, HCC_GZMA, HCC_GPX2, HCC_LTB, HCC_HP, HCC_NTS, and HCC_HRG) were identified from 27 HCC tissues (Fig. 2A,B), and named according to the highest expressed marker genes (Fig. 2C). Among these, we found two cell clusters with obvious sample specificity (HCC_NTS and HCC_HRG). Functional enrichment analysis of each malignant cells cluster showed intratumoral heterogeneity of HCC, such as HCC_NTS and HCC_HRG specifically enriched in Oxidative phosphorylation, Notch signaling pathway, MAPK signaling pathway, and PPAR signaling pathway, suggesting that the enrichment of these pathways contributes to the high heterogeneity of cell clusters (Fig. 2D). Furthermore, we constructed the gene regulatory network (GRN) and found that the GRN with TFs as pivots was organized into two modules (Fig. 2E), the corresponding TFs was shown in Fig. 2F.

The level of cell stemness in various cell clusters was assessed as depicted in Fig. 2G. Score of HCC_FYB is 0.46 ± 0.13 (mean ± STD), HCC_HP is 0.33 ± 0.05, HCC_NTS is 0.31 ± 0.07, and HCC_HRG is 0.31 ± 0.05, significant differences under the Tukey HSD test (*p* < 0.01). In comparison to HCC_NTS and HCC_HRG, HCC_FYB and HCC_HP exhibited a significantly higher degree of stemness. We chose the HCC_FYB subcluster with the highest cell stemness score (Supplementary Table 4) as the initial stage for development and performed pseudo-time analysis on malignant cells. Importantly, we identified HCC_HP that connects HCC_NTS and HCC_HRG (Fig. 2H), which aligns with the cell stemness scoring results. The cell cluster HCC_HP have cells derived from the same sample with HCC_NTS, and also have cells from the same samples with HCC_HRG. HP is mainly expressed in the liver and almost not in other tissues, and downregulated in liver cancer^36^. Our these results suggests that HCC_HP may be malignantly transformed from hepatocytes and is one of the cancer stem cells. Furthermore, we figured out the different HCC subtypes of cell populations between immunotherapy response and immunotherapy-resistant HCC patients (Fig. 2I). It is worth noting that compared to the immunotherapy response group, HCC_HP, HCC_FYB were significantly increased in the immunotherapy-resistant group, and HCC_NTS and HCC_HRG were specifically enriched. These findings further highlight the close correlation between these subgroups and immunotherapy-resistant, which could impact the progression of HCC.

### Stromal cells in HCC exhibit distinct different differentiation trajectories

**Figure 3:**
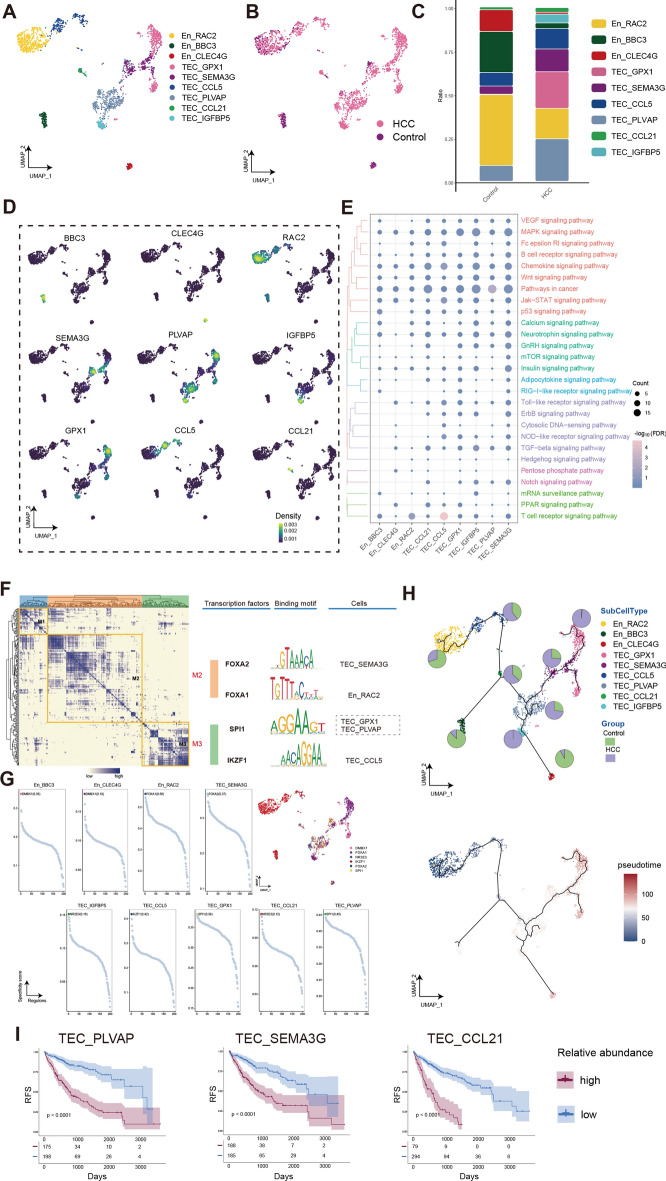
Endothelial cells have heterogeneous differentiation in human HCC. (**A**) Single-cell atlas shows the endothelial cellular clusters of HCC. (**B**) Single-cell atlas shows clusters of endothelial cells in HCC and Control group. (**C**) Differences in the abundance of endothelial cells clusters in HCC tumor tissues and control samples. (**D**) Marker genes for the distinct endothelial cells clusters. Biological pathways in distinct clusters of endothelial cells. (**E**) Bubble colors indicate the significance (−log_10_(FDR)) of enrichment, whereas bubble sizes correspond to the number of genes (Count) enriched in the pathway. (**F**) Transcription factors of endothelial cells clusters in a co-expression pattern. Left: Heat map identified co-expression modules; Middle: major transcription factors and their binding sequences; Right: cell clusters of transcription factors. (**G**) The single-cell atlas showcases the transcription factors regulating gene expression in specific endothelial cells clusters. The scatterplot of each endothelial cells cluster highlights the top-ranked regulon with the highest score. (**H**) Single-cell atlas map the trajectory and pseudotime values of endothelial cells progression. Pie charts show the proportion of the different subpopulations in the clusters. (**I**) Multivariate regression prognostic model for cells clusters of HCC and control, *p* < 0.05 was considered statistically significant. HCC, Hepatocellular carcinoma; En, endothelial cells; TEC, tumor-associated endothelial cells; RFS, relapse-free survival; UMAP, Uniform Manifold Approximation and Projection.

To investigate the ecological landscape heterogeneity of TEC and carcinoma-associated fibroblasts (CAFs) cells in HCC and explore the potential link among stromal cells in HCC tumor progression, we clustered En and Fib cells separately. We applied the UMAP algorithm to cluster the endothelial cells into 9 clusters (Fig. 3A), and then mapped the cell clusters onto groups as shown in Fig. 3B. Several significantly increased tumor endothelial cells (TECs) clusters were observed in the endothelial cells of HCC, such as TEC_PLVAP, TEC_CCL5, TEC_GPX1, and TEC_SEMA3G (Fig. 3C). The highly expressed genes in these endothelial subsets have distinct differences, especially CCL 21, expressed only in a small cluster of cells (Fig. 3D). GO and KEGG enrichment analysis showed that the ErbB signaling pathway and the Notch signaling pathway were upregulated significantly in almost all TEC cell clusters (Fig. 3E). We constructed the GRN and found that the GRN with TFs as pivots was organized into three modules (Fig. 3F), and the specific gene expression was regulated by TFs such as DMBX1, FOXA1, and NR2E3 (Fig. 3G). The pseudotime analysis revealed that En_RAC2 served as the starting point of development throughout the developmental process (Fig. 3H). In contrast, TEC_GPX1 is located at a terminal stage of development, suggesting its fully differentiated state. From the pseudotime analysis we noticed three subpopulations located in the middle state, we constructed a multivariate regression prognostic model for these TECs cells clusters, and found that the relative abundance of them all could be used as great HCC prognostic index (Fig. 3I).

**Figure 4:**
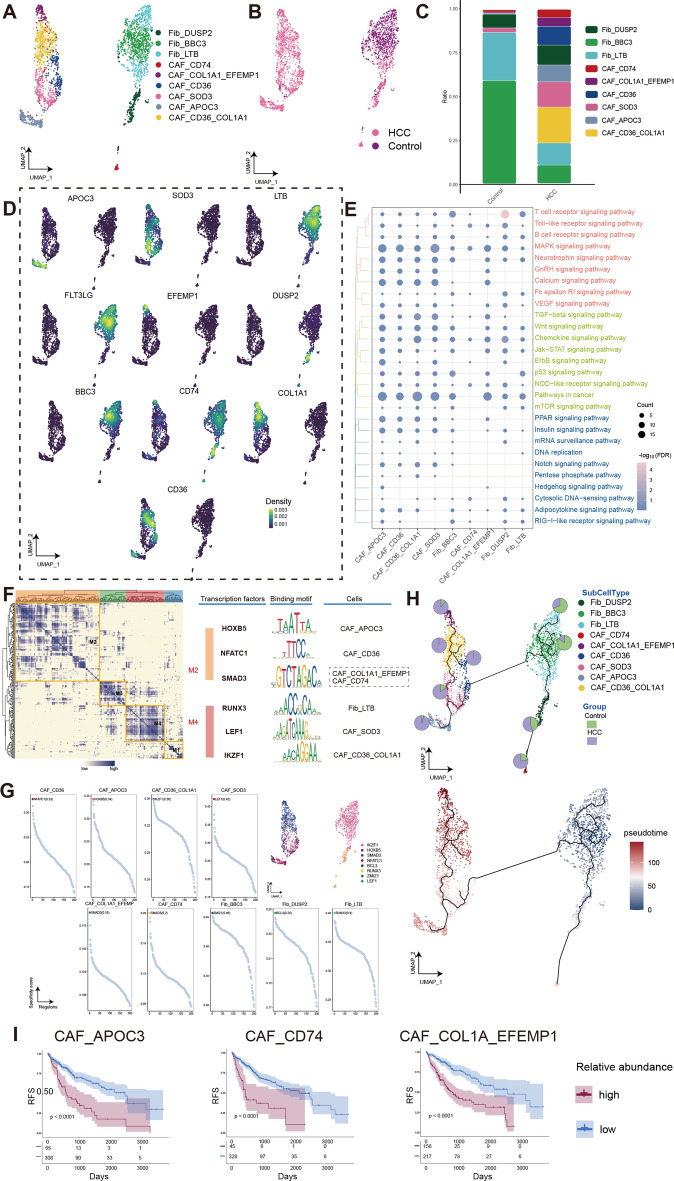
Fibroblasts component in human HCC is highly complex. (**A**) Single-cell atlas shows the fibroblasts cellular clusters of HCC. (**B**) Single-cell atlas shows clusters of fibroblasts in HCC and Control group. (**C**) Differences in the abundance of fibroblasts clusters in HCC tumor tissues and control samples. (**D**) Marker genes for the distinct fibroblasts clusters. (**E**) Biological pathways in distinct clusters of fibroblasts. Bubble colors indicate the significance (−log_10_(FDR)) of enrichment, whereas bubble sizes correspond to the number of genes (Count) enriched in the pathway. (**F**) Transcription factors of fibroblasts clusters in a co-expression pattern. Left: Heat map identified co-expression modules; Middle: major transcription factors and their binding sequences; Right: cell clusters of transcription factors. (**G**) The single-cell atlas showcases the transcription factors regulating gene expression in specific fibroblasts clusters. The scatterplot of each fibroblasts cluster highlights the top-ranked regulon with the highest score. (**H**) Single-cell atlas map the trajectory and pseudotime values of fibroblasts progression. Pie charts show the proportion of the different subpopulations in the clusters. (**I**) Multivariate regression prognostic model for cells clusters of HCC and control, *p* < 0.05 was considered statistically significant. HCC, Hepatocellular carcinoma; Fib, fibroblasts; CAF, carcinoma-associated fibroblasts; RFS, relapse-free survival; UMAP, Uniform Manifold Approximation and Projection.

Additionally, to further explore the associations between Fib and En cells in HCC, we described the single cellular ecological landscape of Fib and also annotated CAFs cells to investigate the communication between TEC and CAF in subsequent studies. We applied the UMAP algorithm to cluster the fibroblasts of BTCs patients into 9 clusters (Fig. 4A,B). Based on the cell proportion, it is evident that CAF cells in HCC are highly complex (Fig. 4C). Two of these cell clusters highly express CD36 and were recently reported to provide an immunosuppressive microenvironment for HCC by secreting macrophage migration inhibitory factors^2^. Markers gene expressions of distinct fibroblasts subpopulations were mapped to the single-cell atlas (Fig. 4D). GO and KEGG enrichment analysis revealed that the ErbB signaling pathway and GnRH signaling pathway were upregulated significantly in almost all CAF cell clusters (Fig. 4E). Based on the GRN, it was found that the GRN with TFs as pivots was organized into four modules (Fig. 4F), and that related TFs regulate the specific gene expression (Fig. 4G). The results of the pseudotime analysis show the developmental differentiation trajectories of CAF. Unlike En, the pseudotemporal developmental process of Fib shows few obvious transition-state cell clusters (Fig. 4H). Therefore, we selected three cell clusters located at the terminal of pseudotime development to construct the multivariate regression prognostic model, evaluated the effect of subpopulations on prognosis. The relative abundance of CAF_APOC3, CAF_CD74 and CAF_COL1A_EFEMP1 all could serves as a potential model for prognostic evaluation (Fig. 4I).

Here, we observed that ErbB signaling pathway is upregulated in both TEC and CAF of HCC, and this pathway is closely related to cell proliferation, migration, differentiation, apoptosis and cell movement, suggesting that the stromal cell activity of HCC is enhanced.

### A new myeloid cell subpopulation were identified

Myeloid-derived cells play an important role in immune checkpoint blockade^37^, where macrophages are among the key cells of TME and play a complex role in tumor development^38^. Neutrophils have recently begun to receive increasing attention in terms of tumor progression suppression, but many aspects are still unknown about neutrophil heterogeneity in tumors^11^.

**Figure 5:**
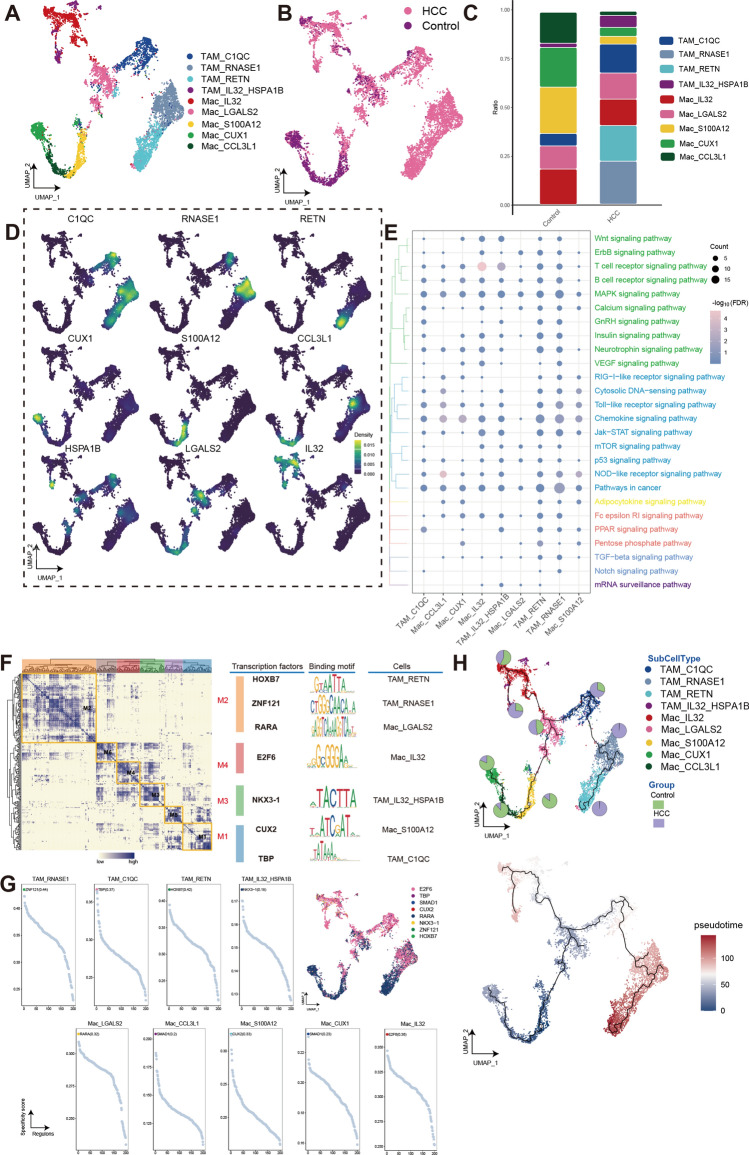
Macrophages have heterogeneous differentiation in human HCC. Single-cell atlas shows the macrophages cellular clusters of HCC. (**A**) Single-cell atlas shows clusters of macrophages in HCC and Control group. (**B**) Differences in the abundance of macrophages clusters in HCC tumor tissues and control samples. (**C**) Marker genes for the distinct macrophages clusters. (**D**) Biological pathways in distinct clusters of macrophages. Bubble colors indicate the significance (−log_10_(FDR)) of enrichment, whereas bubble sizes correspond to the number of genes (Count) enriched in the pathway. (**E**) Transcription factors of macrophages clusters in a co-expression pattern. Left: Heat map identified co-expression modules; Middle: major transcription factors and their binding sequences; Right: cell clusters of transcription factors. (**F**) The single-cell atlas showcases the transcription factors regulating gene expression in specific macrophages clusters. The scatterplot of each macrophages cluster highlights the top-ranked regulon with the highest score. (**G**) Single-cell atlas map the trajectory and pseudotime values of macrophages progression. Pie charts show the proportion of the different subpopulations in the clusters. HCC, Hepatocellular carcinoma; Mac, macrophages; TAM, tumor-associated macrophages; UMAP, Uniform Manifold Approximation and Projection.

**Figure 6:**
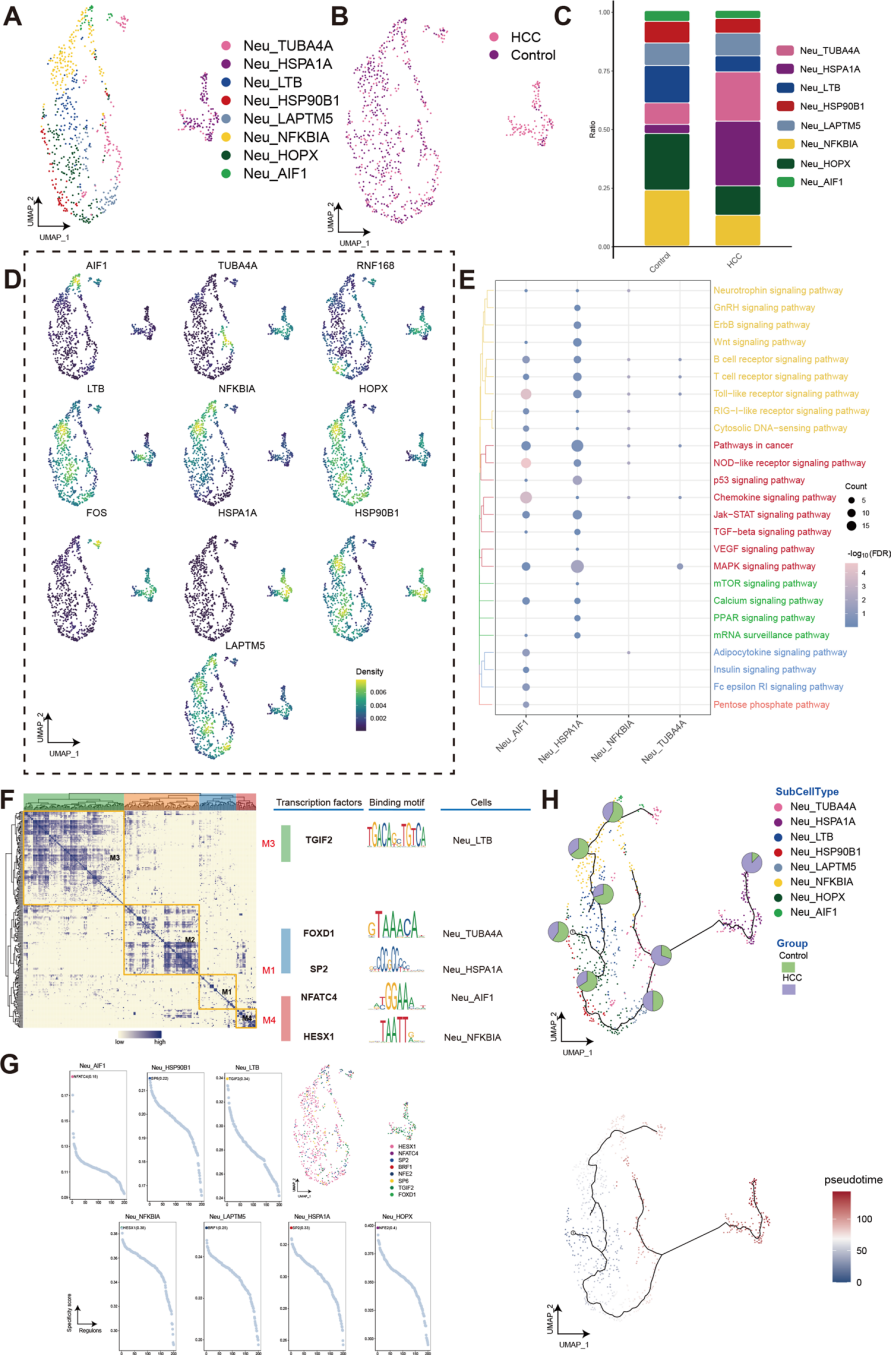
Neutrophils (Neu) have heterogeneous differentiation in human HCC. (**A**) Single-cell atlas shows the neutrophils cellular clusters of HCC. (**B**) Single-cell atlas shows clusters of neutrophils in HCC and Control group. (**C**) Differences in the abundance of neutrophils clusters in HCC tumor tissues and control samples. (**D**) Marker genes for the distinct neutrophils clusters. (**E**) Biological pathways in distinct clusters of neutrophils. Bubble colors indicate the significance (−log_10_(FDR)) of enrichment, whereas bubble sizes correspond to the number of genes (Count) enriched in the pathway. (**F**) Transcription factors of neutrophils clusters in a co-expression pattern. Left: Heat map identified co-expression modules; Middle: major transcription factors and their binding sequences; Right: cell clusters of transcription factors. (**G**) The single-cell atlas showcases the transcription factors regulating gene expression in specific neutrophils clusters. The scatterplot of each neutrophils cluster highlights the top-ranked regulon with the highest score. (**H**) Single-cell atlas map the trajectory and pseudotime values of neutrophils progression. Pie charts show the proportion of the different subpopulations in the clusters. HCC, Hepatocellular carcinoma; Neu, Neutrophils; UMAP, Uniform Manifold Approximation and Projection.

To characterize myeloid immune cells in HCC, we reclustered macrophages (into 9 clusters) and neutrophils (into 8 clusters) (Figs. 5A,B and 6A,B). The TAM components of HCC reflects its high plasticity and are divided into four TAM subtypes (Fig. 5C), the expression of marker genes in distinct cell clusters shown in Fig. 5D, GnRH signaling pathway and ErbB signaling pathway was upregulated in TAM of HCC (Fig. 5E). The GRN with TFs as the pivots was organized into six modules (Fig. 5F) and regulated by eight TFs (Fig. 5G). The results of the pseudotime analysis shows different differentiation trajectory in TAM of HCC (Fig. 5H). Specifically, TAM_IL32_HSPA1B arises through the differentiation of Mac_IL32, while TAM_C1QC gives rise to TAM_RANASE through further differentiation, which is facilitated by the participation of Mac_LGALS2.

Two significantly increased cell clusters (Neu_HSPA1A and Neu_TUBA4A) were identified in HCC (Fig. 6C). Moreover, three cell clusters (Neu_Neu LTB, Neu_HOPX, and Neu_NFKB1A) showed significant decreases in HCC. Markers of distinct neutrophils subpopulations were mapped to the single-cell atlas (Fig. 6D). Same as TAM cells, GnRH signaling pathway and ErbB signaling pathway are upregulated in the HCC related cell cluster Neu_HSPA1A (Fig. 6E). The GRN with TFs as the pivots was organized into four modules (Fig. 6F). These TFs (TGIF2, FOXD1, SP2, NFATC4, and HESX1) regulate the specific gene expression (Fig. 6G). The results of the pseudotime analysis show that Neu_HOPX serves as the starting point for development, evolving into Neu_HSP90B1, Neu_LTB, and Neu_AIF1 subclusters (Fig. 6H). Additionally, Neu_HSPA1A is a developmental differentiation terminal cluster.

**Figure 7:**
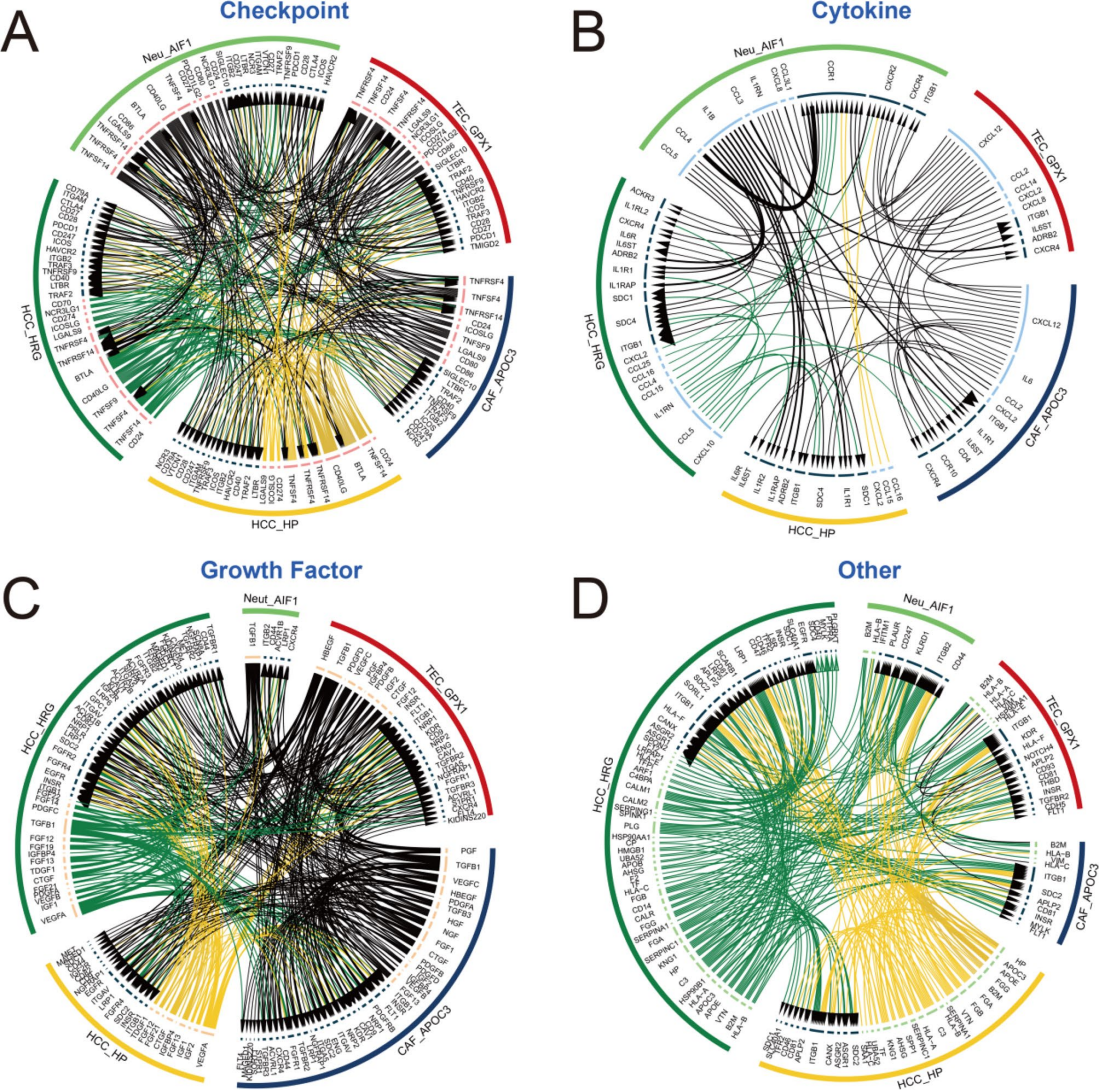
The receptor-ligand pairs among HP gene positive expression cells. (**A**) The immune checkpoint receptor-ligand pairs among HP gene positive expression cells. (**B**) The chemokine receptor-ligand pairs among HP gene positive expression cells. (**C**) The growth factor receptor-ligand pairs among HP gene positive expression cells. (**D**) The other receptor-ligand pairs among HP gene positive expression cells.

In tumor cells, we found a subtype of interest HCC_HP, and analyzed HP positive cell type, observing a cluster of neutrophils (Neu_AIF1) that showed positive expression of the HP gene. To explore the intercellular communication between this neutrophil subset and the HCC_HP, we performed the iTALK analysis (Fig. 7). These HP positive clusters (Neu_AIF1, CAF_APOC3, TEC_GPX1, HCC_HRG and HCC_HP) demonstrate extremely extensive intercellular communication. These results suggest that we may be able to inhibit HCC tumor progression by removing this newly identified neutrophil cluster (Neu_AIF1).

### Lymphocytes in HCC are reprogrammed by the tumors

**Figure 8:**
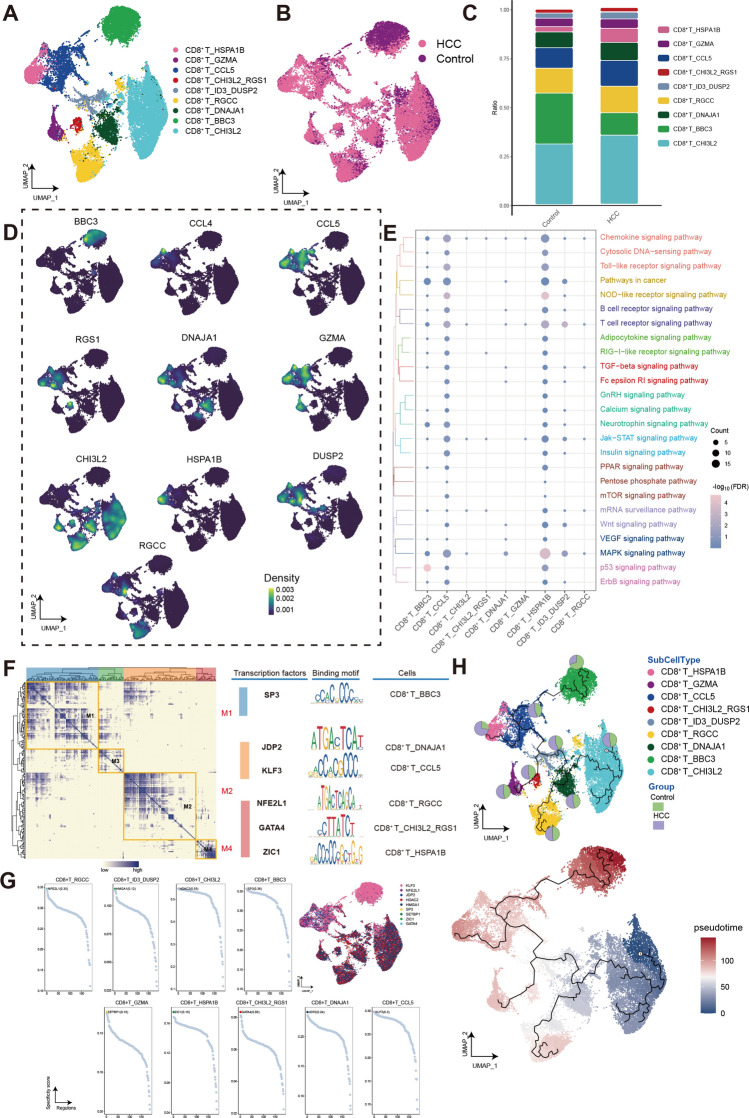
CD8^+^ T cells have heterogeneous differentiation in human HCC. (**A**) Single-cell atlas shows the CD8^+^ T cellular clusters of HCC. (**B**) Single-cell atlas shows clusters of CD8^+^ T cells in HCC and Control group. (**C**) Differences in the abundance of CD8^+^ T cells clusters in HCC tumor tissues and control samples. (**D**) Marker genes for the distinct CD8^+^ T cells clusters. (**E**) Biological pathways in distinct clusters of CD8^+^ T cells. Bubble colors indicate the significance (−log_10_(FDR)) of enrichment, whereas bubble sizes correspond to the number of genes (Count) enriched in the pathway. (**F**) Transcription factors of CD8^+^ T cells clusters in a co-expression pattern. Left: Heat map identified co-expression modules; Middle: major transcription factors and their binding sequences; Right: cell clusters of transcription factors. (**G**) The single-cell atlas showcases the transcription factors regulating gene expression in specific CD8^+^ T cells clusters. The scatterplot of each CD8^+^ T cells cluster highlights the top-ranked regulon with the highest score. (**H**) Single-cell atlas map the trajectory and pseudotime values of CD8^+^ T cells progression. Pie charts show the proportion of the different subpopulations in the clusters. HCC, Hepatocellular carcinoma; UMAP, Uniform Manifold Approximation and Projection.

T-lymphocytes play an important effector role in killing in anti-tumor immunity. Here, we characterize the single-cell landscape of CD8^+^ T cells of HCC. The CD8^+^ T cell clusters of HCC were re-clustered to obtain 9 cell clusters (Fig. 8A,B). Interestingly, the one of the CD8^+^ T clusters with high HSPA1B expression (CD8^+^ T_HSPA1B) was significantly higher in HCC compared to the control (Fig. 8C,D). Similar to myeloid cells, we observed that the GnRH signaling pathway and ErbB signaling pathway pathway were upregulated in these two HCC associated CD8^+^ T cell clusters (Fig. 8E), however, both signaling pathways are downregulated in HCC-related NKT cell (Supplementary Fig. 1E). The GRN with TFs as pivots was organized into four modules (Fig. 8F) and regulated by night TFs (Fig. 8G). The results of the pseudotime analysis also indicate that CD8^+^ T_HSPA1B is at a developmental terminal, indicating that it is in a fully differentiated state (Fig. 8H).

**Figure 9:**
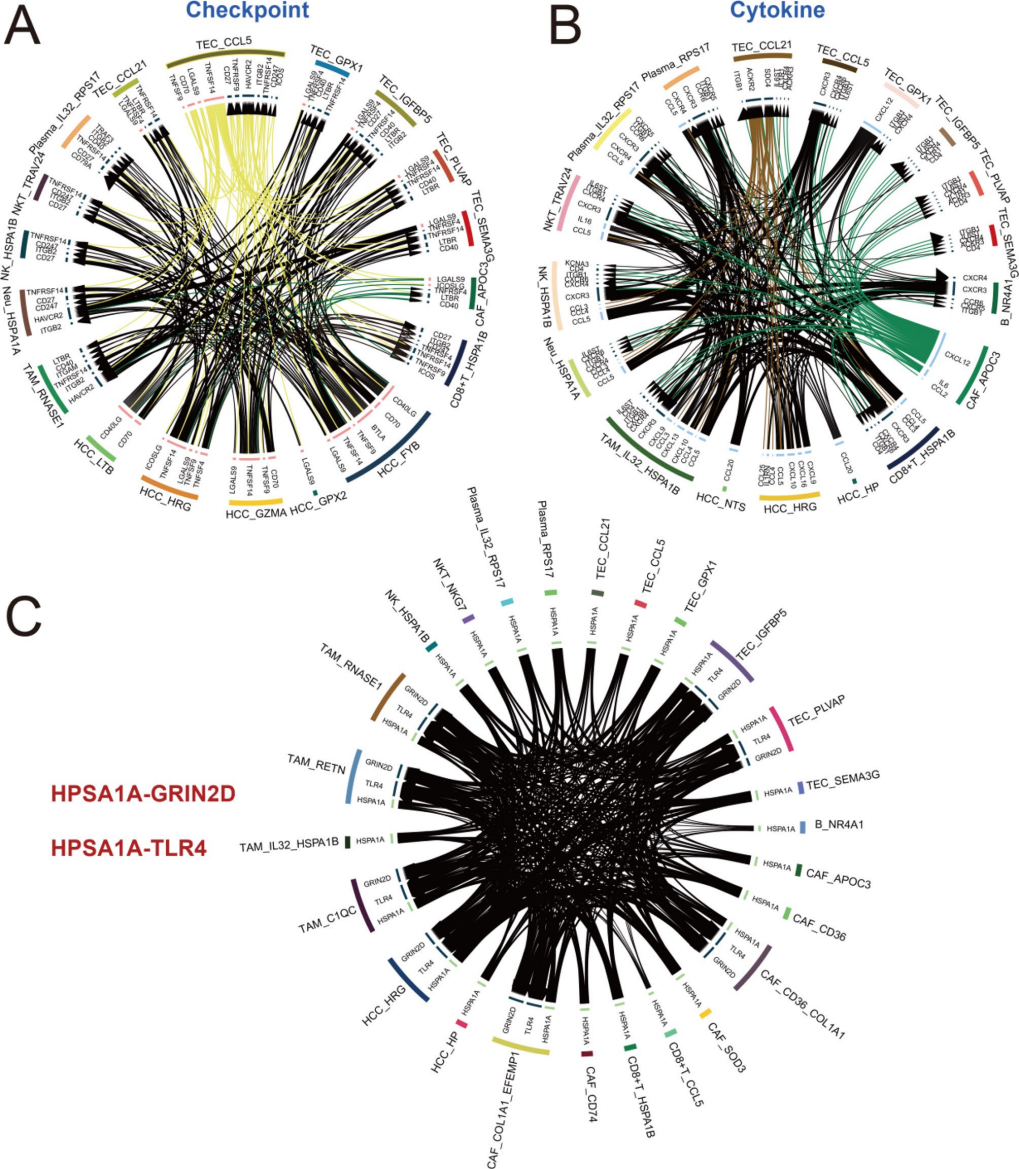
The receptor-ligand pairs among HCC tumor cells and TME cells. (**A**) The immune checkpoint receptor-ligand pairs among HCC tumor cells and TME cells. (**B**) The chemokine receptor-ligand pairs among HCC tumor cells and TME cells. (**C**) The receptor-ligand pairs among HSPA1 positive cells.

We observed that expression of HSPA1B was found not only in T lymphocytes, but also in NK lymphocytes (Supplementary Fig. 2). We speculated that malignant cells in HCC might be undergo immune reprogramming with HSPA1 as the axis, so we conducted intercellular communication analysis for all cell clusters that were positive for HSPA1 (HSPA1A or HSPA1B). Consistent with our speculation, there is extensive and complex intercellular communication between tumor cells and all HSPA1 positive cell clusters, suggesting that TME cells in HCC may be metabolically reprogrammed by tumor cells via the HSPA1-GRIN2D/TLR4 axis to promote tumor progression (Fig. 9).

## Discussion

Immunotherapy has promising prospects for the treatment of advanced HCC, but the current response rates are low, which is closely related to the composition of the tumor and TME^39^. Here, we provide a global single-cell transcriptome atlas to characterize the tumor ecosystem in HCC, performed a comprehensive analysis of the malignant cells, stromal cells, myeloid cells, and lymphocytes of TME in HCC, also constructed a prognostic model for some of these stromal cells.

Targeted therapies are likely to induce the stemness transformation of cancer cells and acquire drug resistance^9,40^. Stemness score is an indicator to assess the similarity between tumor cells and stem cells, which is associated with the active biological processes in stem cells as well as the higher degree of tumor dedifferentiation^41^. Our analysis revealed the presence of a malignant cell cluster (HCC_HP) in HCC that may contribute to the heterogeneity of the tumor, HP is downregulated in human HCC, the cell cluster HCC_HP have cells derived from the same sample with HCC_NTS, and also have cells from the same samples with HCC_HRG, possessed a highly stemness score, be located in a key position at the differentiation trajectory, which may possible differentiate into highly heterogeneous malignant cell subtypes (e.g., HCC_HRG and HCC_NTS). Additionlly, HCC_HRG and HCC_NTS exhibit significant enrichment within the Oxidative phosphorylation, MAPK signaling pathway, and the PPAR signaling pathway. Previous research has indicated that the NTS potentially regulates the MAPK signaling pathway, thereby facilitating HCC progression^42^. The PPAR signaling pathway potentially plays a role in influencing the targeted induction of HCC differentiation in cancer stem cells^43^. Notably, the HCC_HP subcluster exhibits no enrichment in pathways related to oxidative phosphorylation. This observation might account for the complete loss of gene expression related to oxidative phosphorylation within the population of HCC_HP.

The main focus of cancer immunotherapy is to interrupt the suppression of anti-tumor lymphocytes. In addition to lymphocytes, the HCC environment also includes many other immune cell types, such as neutrophils are becoming an important contributor to the pathogenesis of HCC, several neutrophils for HCC patients is currently in clinical trials^10^. We found that Neu_HOPX, as the starting point of the developmental trajectory, may play an important role in neutrophil differentiation. Based on the intercellular ligand receptor interaction analysis of cell clusters with HP positive expression, indicating the active crosstalk between these cells. A novel cell cluster (Neu_AIF1) of immune cells was identified from these clusters and might hold value in immunotherapy research. Previous studies have demonstrated abnormal expression of LTB in human HCC samples, suggesting its potential as a novel target for HCC^44^. Moreover, we also discovered its expression in neutrophils, and the precise role of Neu_LTB requires additional investigation.

We observed that HSPA1B was specifically expressed not only in T lymphocytes, but also NK lymphocytes (Supplementary Fig. 2). We speculated that malignant cells in HCC might undergo immune reprogramming with HSPA1 as the axis, so we conducted intercellular communication analysis for all cell clusters with positive expression of HSPA1 (HSPA1A or HSPA1B). Consistent with our speculation, extensive and complex intercellular communication exists between tumor cells and all HSPA1-positive cell clusters. We hypothesized that tumor cells may metabolically reprogram TME cells in HCC through the HSPA1-GRIN2D/TLR4 axis to promote tumor progression (Fig. 9).

In the global landscape analysis of TME of HCC, we found that many subsets of different cell types highly expressed the heat shock protein HSPA1 related gene (HSPA1A and HSPA1B), we speculate that there may be a metabolic reprogramming associated with HSPA1 in HCC. Accordingly, we performed intercellular communication analysis of HSPA1 positive expression cell clusters in all cell types, and found that hyperactive crosstalk between HCC associated TECs, CAFs, TAM, neutrophils, plasma cells, NK cells, NKT cells, and CD8^+^ T cells. The cell cluster (TEC_CCL21) interacts with almost all immune cells, and CCL21 has been shown to promote tumor immune escape^45,46^. Meanwhile, in the enrichment analysis of these cells, we noted the upregulation of ErbB signaling pathway, this pathway was directly related to exosome-mediated signal transduction in tumors^47,48^. In summary, we hypothesized that exosome-mediated metabolic reprogramming with the HSPA1-GRIN2D/TLR4 axis appeared in HCC, possibly use as a important target for immunotherapy research of HCC. However, due to the limited availability of HCC data, we can only mine the limited biological information in less than 30 samples, many HCC malignant cells and TME cell types with higher heterogeneity still need to obtain more sequencing samples to understand, more samples of HCC could be considered for analysis in the future to assist in clinical treatment studies.

## Conclusion

Our study analyzed the single-cell landscape heterogeneity of human HCC, identified new cell clusters in tumor cells (HCC_HP) and neutrophils (Neu_AIF1) with implications for immunotherapy research, discovered complex intercellular communication between tumor cells and TME cells, and established high confidence prognostic model. We speculated from the results of the significantly up-regulated signaling pathway (ErbB signaling pathway) and HSPA1-positive multicellular communication analysis that there is an exosome-mediated metabolic reprogramming process in HCC, which provides a meaningful target reference for immunotherapy studies.

### Supplementary Information

Supplementary Figure S1.

Supplementary Figure S2.

Supplementary Figure S3.

Supplementary Table S1.

Supplementary Table S2.

Supplementary Table S3.

Supplementary Table S4.

## Data Availability

All data analysed during this study are included in this published article.

## Abbreviations

HCC: Hepatocellular carcinoma
TME: Tumor microenvironment
TECs: Tumor endothelial cells
CAFs: Cancer-associated fibroblasts
CSC: Cancer stem cell-like cells
scRNA-seq: Single-cell RNA sequencing
QC: Quality control
UMAP: Uniform manifold approximation and projection
DEGs: Differentially expressed genes
TCGA: The cancer genome atlas
CNVs: Copy number variants
KEGG: Kyoto encyclopedia of genes and genomes
TFs: Transcription factors
OS: Overall survival
RFS: Relapse-free survival
GRN: Gene regulatory network
