# Supplementary material for: Single-cell RNA sequencing revealed potential targets for immunotherapy studies in hepatocellular carcinoma
Source: Sci Rep. 2023 Nov 1;13:18799. doi: 10.1038/s41598-023-46132-w (PMC10620237; doi:10.1038/s41598-023-46132-w)
Supplement: Supplementary file 1 — Supplementary Figure S1. [file 41598_2023_46132_MOESM1_ESM.pdf]

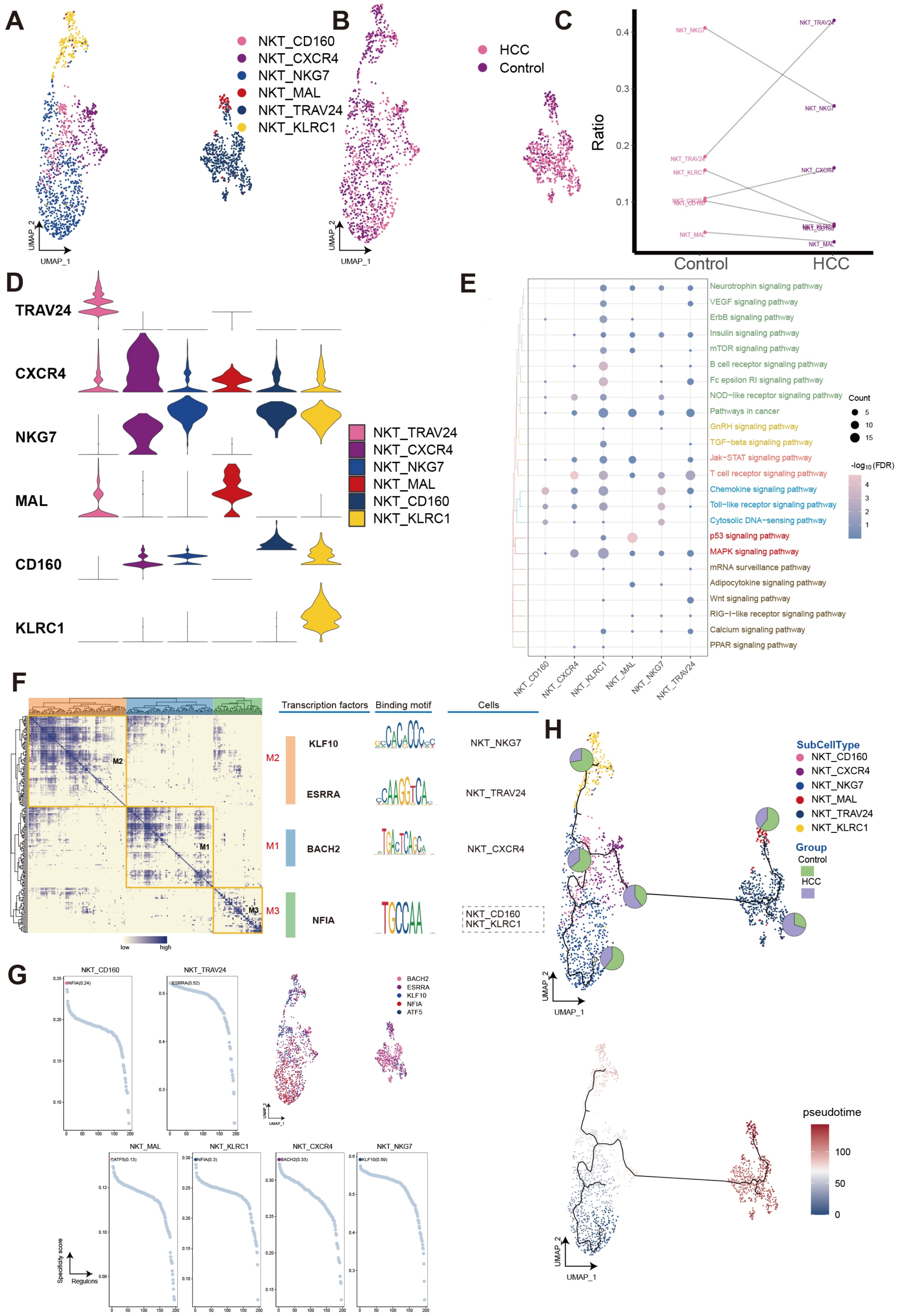

Supplementary Figure 1. NKT cells have heterogeneous differentiation in human HCC

(A) Single-cell atlas shows the NKT cellular clusters of HCC.

(B) Single-cell atlas shows clusters of NKT cells in HCC and Control group.

(C) Differences in the abundance of NKT cells clusters in HCC tumor tissues and control samples.

(D) Violin plots showing marker genes for the distinct NKT cells clusters.

(E) Biological pathways in distinct clusters of NKT cells. Bubble colors indicate the significance ($-\log_{10}$(FDR)) of enrichment, whereas bubble sizes correspond to the number of genes (Count) enriched in the pathway.

(F) Transcription factors of NKT cells clusters in a co-expression pattern. Left: Heat map identified co-expression modules; Middle: major transcription factors and their binding sequences; Right: cell clusters of transcription factors.

(G) The single-cell atlas showcases the transcription factors regulating gene expression in specific NKT cells clusters. The scatterplot of each NKT cells cluster highlights the top-ranked regulon with the highest score.

(H) Single-cell atlas map the trajectory and pseudotime values of NKT cells progression. Pie charts show the proportion of the different subpopulations in the clusters.

HCC, Hepatocellular carcinoma; UMAP, Uniform Manifold Approximation and Projection.
