# Supplementary material for: Single-cell RNA sequencing revealed potential targets for immunotherapy studies in hepatocellular carcinoma
Source: Sci Rep. 2023 Nov 1;13:18799. doi: 10.1038/s41598-023-46132-w (PMC10620237; doi:10.1038/s41598-023-46132-w)
Supplement: Supplementary file 3 — Supplementary Figure S3. [file 41598_2023_46132_MOESM3_ESM.pdf]

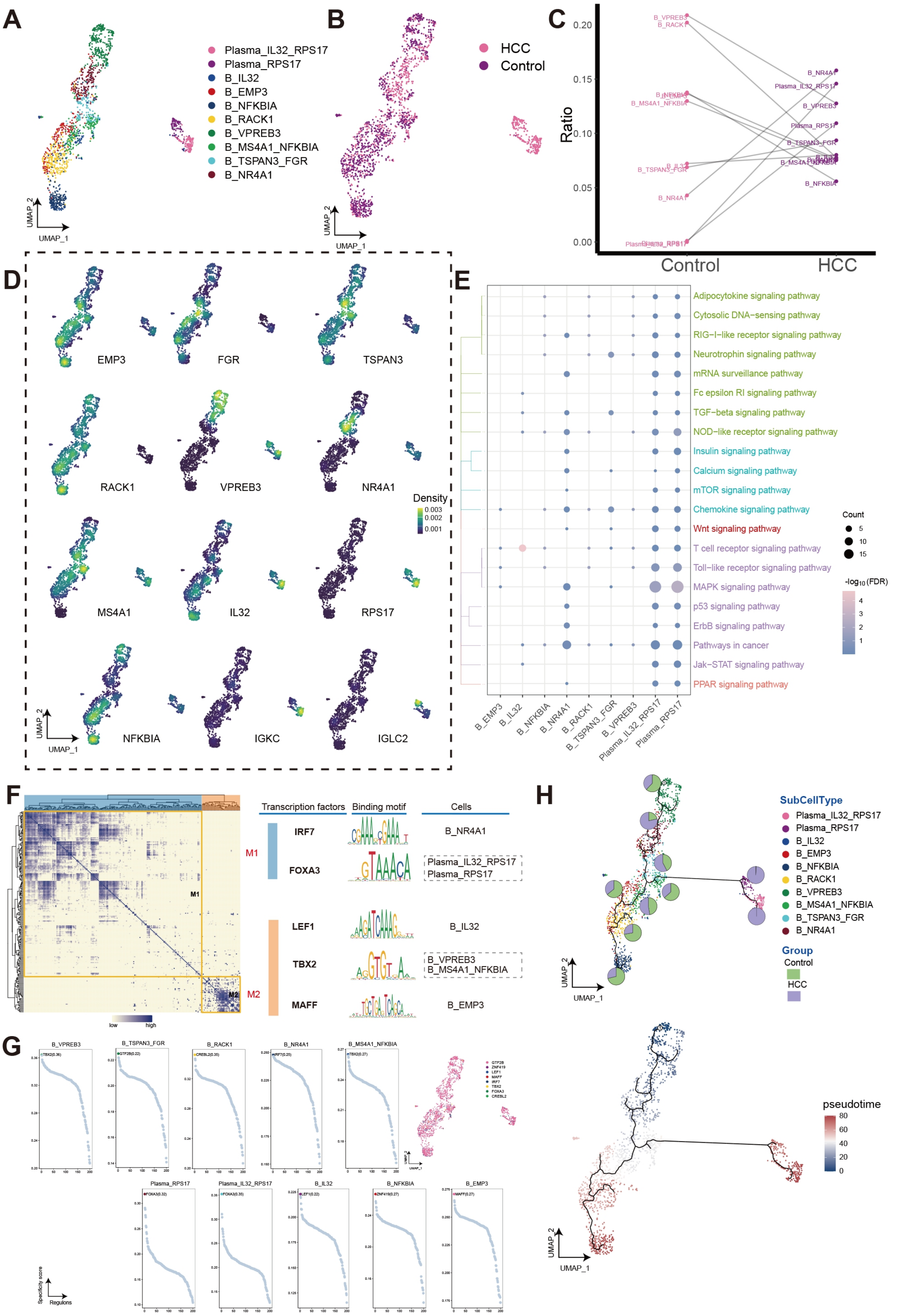

Supplementary Figure 3. B cells have heterogeneous differentiation in human HCC

(A) Single-cell atlas shows the B cellular clusters of HCC.

(B) Single-cell atlas shows clusters of B cells in HCC and Control group.

(C) Differences in the abundance of B cells clusters in HCC tumor tissues and control samples.

(D) Marker genes for the distinct B cells clusters.

(E) Biological pathways in distinct clusters of B cells. Bubble colors indicate the significance ($-\log_{10}$(FDR)) of enrichment, whereas bubble sizes correspond to the number of genes (Count) enriched in the pathway.

(F) Transcription factors of B cells clusters in a co-expression pattern. Left: Heat map identified co-expression modules; Middle: major transcription factors and their binding sequences; Right: cell clusters of transcription factors.

(G) The single-cell atlas showcases the transcription factors regulating gene expression in specific B cells clusters. The scatterplot of each B cells cluster highlights the top-ranked regulon with the highest score.

(H) Single-cell atlas map the trajectory and pseudotime values of B cells progression. Pie charts show the proportion of the different subpopulations in the clusters.

Plasma, Plasma cells; HCC, Hepatocellular carcinoma; UMAP, Uniform Manifold Approximation and Projection.
