# Supplementary material for: Single-cell RNA sequencing revealed potential targets for immunotherapy studies in hepatocellular carcinoma
Source: Sci Rep. 2023 Nov 1;13:18799. doi: 10.1038/s41598-023-46132-w (PMC10620237; doi:10.1038/s41598-023-46132-w)
Supplement: Supplementary file 4 — Supplementary Table S1. [file 41598_2023_46132_MOESM4_ESM.pdf]

Supplementary Table 1. HCC-related single-cell RNA sequencing (scRNA-seq) data

Data sources	Platform ID	The single-cell library preparation platforms
GSE125449	GPL18573	Illumina NextSeq 500 (Homo sapiens)
GSE125449	GPL18573	Illumina NextSeq 500 (Homo sapiens)
GSE125449	GPL18573	Illumina NextSeq 500 (Homo sapiens)
GSE125449	GPL18573	Illumina NextSeq 500 (Homo sapiens)
GSE125449	GPL18573	Illumina NextSeq 500 (Homo sapiens)
GSE125449	GPL18573	Illumina NextSeq 500 (Homo sapiens)
GSE125449	GPL18573	Illumina NextSeq 500 (Homo sapiens)
GSE125449	GPL20301	Illumina HiSeq 4000 (Homo sapiens)
GSE125449	GPL20301	Illumina HiSeq 4000 (Homo sapiens)
GSE210679	GPL20795	HiSeq X Ten (Homo sapiens)
GSE189903	GPL24676	Illumina NovaSeq 6000 (Homo sapiens)
GSE189903	GPL24676	Illumina NovaSeq 6000 (Homo sapiens)
GSE189903	GPL24676	Illumina NovaSeq 6000 (Homo sapiens)
GSE189903	GPL24676	Illumina NovaSeq 6000 (Homo sapiens)
GSE189903	GPL24676	Illumina NovaSeq 6000 (Homo sapiens)
GSE189903	GPL24676	Illumina NovaSeq 6000 (Homo sapiens)
GSE189903	GPL24676	Illumina NovaSeq 6000 (Homo sapiens)
GSE189903	GPL24676	Illumina NovaSeq 6000 (Homo sapiens)
GSE189903	GPL24676	Illumina NovaSeq 6000 (Homo sapiens)
GSE189903	GPL24676	Illumina NovaSeq 6000 (Homo sapiens)
GSE189903	GPL24676	Illumina NovaSeq 6000 (Homo sapiens)
GSE189903	GPL24676	Illumina NovaSeq 6000 (Homo sapiens)
GSE189903	GPL24676	Illumina NovaSeq 6000 (Homo sapiens)
GSE189903	GPL24676	Illumina NovaSeq 6000 (Homo sapiens)
GSE189903	GPL24676	Illumina NovaSeq 6000 (Homo sapiens)
GSE189903	GPL24676	Illumina NovaSeq 6000 (Homo sapiens)
GSE189903	GPL24676	Illumina NovaSeq 6000 (Homo sapiens)
GSE189903	GPL24676	Illumina NovaSeq 6000 (Homo sapiens)
GSE189903	GPL24676	Illumina NovaSeq 6000 (Homo sapiens)
GSE189903	GPL24676	Illumina NovaSeq 6000 (Homo sapiens)
GSE189903	GPL24676	Illumina NovaSeq 6000 (Homo sapiens)

The sequencing platforms	Data Types	Sample	The number of cells	Group
10x Genomics platform	mtx	GSM4050085	124	HCC
10x Genomics platform	mtx	GSM4050086	704	HCC
10x Genomics platform	mtx	GSM4050087	151	HCC
10x Genomics platform	mtx	GSM4050090	124	HCC
10x Genomics platform	mtx	GSM4050092	805	HCC
10x Genomics platform	mtx	GSM4050094	132	HCC
10x Genomics platform	mtx	GSM4050095	1046	HCC
10x Genomics platform	mtx	GSM4050098	238	HCC
10x Genomics platform	mtx	GSM4050108	589	HCC
10x Genomics	mtx	GSM6435354	9439	HCC
10x Genomics	mtx	GSM5709316	11344	Control
10x Genomics	mtx	GSM5709321	110	Control
10x Genomics	mtx	GSM5709324	8769	Control
10x Genomics	mtx	GSM5709329	10574	Control
10x Genomics	mtx	GSM5709314	8991	HCC
10x Genomics	mtx	GSM5709315	8130	HCC
10x Genomics	mtx	GSM5709317	1509	HCC
10x Genomics	mtx	GSM5709318	800	HCC
10x Genomics	mtx	GSM5709319	1190	HCC
10x Genomics	mtx	GSM5709320	6103	HCC
10x Genomics	mtx	GSM5709322	969	HCC
10x Genomics	mtx	GSM5709323	969	HCC
10x Genomics	mtx	GSM5709325	817	HCC
10x Genomics	mtx	GSM5709326	6401	HCC
10x Genomics	mtx	GSM5709327	2249	HCC
10x Genomics	mtx	GSM5709328	2173	HCC
10x Genomics	mtx	GSM5709330	1450	HCC
10x Genomics	mtx	GSM5709331	2132	HCC
10x Genomics	mtx	GSM5709332	2109	HCC
10x Genomics	mtx	GSM5709336	7141	HCC
10x Genomics	mtx	GSM5709337	1052	HCC
