# Supplementary material for: Single-cell RNA sequencing revealed potential targets for immunotherapy studies in hepatocellular carcinoma
Source: Sci Rep. 2023 Nov 1;13:18799. doi: 10.1038/s41598-023-46132-w (PMC10620237; doi:10.1038/s41598-023-46132-w)
Supplement: Supplementary file 5 — Supplementary Table S2. [file 41598_2023_46132_MOESM5_ESM.pdf]

Supplementary Table 2. Cell markers used for cell clustering and annotation

CellMarker	CellType
HSPA5	HCC
MKI67	HCC
BIRC5	HCC
GLUL	HCC
KRT7	HCC
ARG1	HCC
BSG	HCC
CDKN2A	HCC
KRT19	HCC
EPCAM	HCC
MUC1	HCC
GPC3	HCC
AFP	HCC
CHI3L1	HCC
ENG	En
PECAM1	En
CD34	En
CDH5	En
VWF	En
KDR	En
ICAM1	En
TEK	En
VCAM1	En
ITGB1	Fibroblasts
S100A4	Fibroblasts
ACTA2	Fibroblasts
PDGFRB	Fibroblasts
COL1A1	Fibroblasts
CD81	Fibroblasts
LRP1	Fibroblasts
CD8A	CD8.T
CD8B	CD8.T
CD3D	CD8.T
CD3E	CD8.T
CD3G	CD8.T
IFNG	CD8.T
CD247	CD8.T
PTPRC	CD8.T
CD160	NKT
CD247	NKT
PTPRC	NKT
TRAV24	NKT

CD3D	NKT
CD3G	NKT
CD3E	NKT
PTPRC	B
CD79A	B
CD79B	B
MS4A1	B
CD19	B
CD22	B
CD70	B
CD1A	MDDC
CD1C	MDDC
PTPRC	Neutrophils
FCGR3A	Neutrophils
FCGR3B	Neutrophils
CLEC4C	pDC
IL3RA	pDC
CD244	NK
PTPRC	NK
EOMES	NK
TCF7	ILC
IL7R	ILC
IL2RA	ILC
ID2	ILC
NCR1	ILC
RUNX3	ILC
PTPRC	ILC
PTPRC	Naive.T
CD3D	Naive.T
CD3E	Naive.T
CD3G	Naive.T
CD247	Naive.T
CD24	Ep
EPCAM	Ep
ITGA6	Ep
CD68	Mac
CD14	Mac
PTPRC	Mac
