# Supplementary material for: Single-cell RNA sequencing revealed potential targets for immunotherapy studies in hepatocellular carcinoma
Source: Sci Rep. 2023 Nov 1;13:18799. doi: 10.1038/s41598-023-46132-w (PMC10620237; doi:10.1038/s41598-023-46132-w)
Supplement: Supplementary file 6 — Supplementary Table S3. [file 41598_2023_46132_MOESM6_ESM.pdf]

Supplementary Table 3. Cell markers of different cell clusters

Clusters	CellMarkers	CellTypes
0	PTPRC	CD8.T
0	CD3D	CD8.T
0	CD3E	CD8.T
0	CD3G	CD8.T
0	CD247	CD8.T
0	CD8A	CD8.T
0	CD8B	CD8.T
1	PTPRC	CD8.T
1	CD3D	CD8.T
1	CD3E	CD8.T
1	CD3G	CD8.T
1	CD247	CD8.T
1	CD8A	CD8.T
1	CD8B	CD8.T
2	PTPRC	Mac
2	CD14	Mac
2	CD68	Mac
3	PTPRC	Naive.T
3	CD3D	Naive.T
3	CD3E	Naive.T
3	CD3G	Naive.T
4	PTPRC	Naive.T
4	CD3D	Naive.T
4	CD3E	Naive.T
4	CD3G	Naive.T
4	CD247	Naive.T
5	PTPRC	Naive.T
5	CD3D	Naive.T
5	CD3E	Naive.T
5	CD3G	Naive.T
5	CD247	Naive.T
6	PTPRC	CD8.T
6	CD3D	CD8.T
6	CD3E	CD8.T
6	CD3G	CD8.T
6	CD247	CD8.T
6	CD8A	CD8.T
6	CD8B	CD8.T
7	PTPRC	CD8.T
7	CD3D	CD8.T
7	CD3E	CD8.T
7	CD3G	CD8.T

7	CD247	CD8.T
7	CD8A	CD8.T
7	CD8B	CD8.T
8	PTPRC	Mac
8	CD68	Mac
9	PTPRC	Naive.T
9	CD3D	Naive.T
9	CD3E	Naive.T
9	CD3G	Naive.T
9	CD247	Naive.T
10	PTPRC	DoubleCell
10	CD79A	DoubleCell
10	CD3D	DoubleCell
10	CD3E	DoubleCell
10	CD3G	DoubleCell
10	CD247	DoubleCell
10	CD8A	DoubleCell
10	CD8B	DoubleCell
11	PTPRC	ILC
11	IL2RA	ILC
12	PTPRC	DoubleCell
12	CD79B	DoubleCell
12	CD3D	DoubleCell
12	CD3E	DoubleCell
12	CD3G	DoubleCell
12	CD247	DoubleCell
13	PTPRC	Naive.T
13	CD3D	Naive.T
13	CD3E	Naive.T
13	CD3G	Naive.T
13	CD247	Naive.T
14	PTPRC	CD8.T
14	CD3D	CD8.T
14	CD3E	CD8.T
14	CD3G	CD8.T
14	CD247	CD8.T
14	CD8A	CD8.T
14	CD8B	CD8.T
14	IFNG	CD8.T
15	PTPRC	Naive.T
15	CD3D	Naive.T
15	CD3E	Naive.T
15	CD3G	Naive.T
15	CD247	Naive.T
16	PTPRC	Naive.T
16	CD3D	Naive.T

16	CD3E	Naive.T
16	CD3G	Naive.T
16	CD247	Naive.T
17	MKI67	HCC
17	BIRC5	HCC
17	HSPA5	HCC
18	CD19	B
18	CD22	B
18	CD79A	B
18	CD79B	B
18	MS4A1	B
19	PTPRC	NK
19	CD247	NK
19	CD244	NK
19	EOMES	NK
20	BSG	HCC
20	MKI67	HCC
21	ACTA2	Fibroblasts
22	PTPRC	CD8.T
22	CD3D	CD8.T
22	CD3E	CD8.T
22	CD3G	CD8.T
22	CD247	CD8.T
22	CD8A	CD8.T
22	CD8B	CD8.T
23	ACTA2	Fibroblasts
23	PDGFRB	Fibroblasts
23	ITGB1	Fibroblasts
23	COL1A1	Fibroblasts
23	CD81	Fibroblasts
23	LRP1	Fibroblasts
24	ENG	En
24	PECAM1	En
24	CD34	En
24	CDH5	En
24	VWF	En
24	KDR	En
24	ICAM1	En
24	TEK	En
25	PTPRC	NKT
25	CD3D	NKT
25	CD3E	NKT
25	CD3G	NKT
25	CD247	NKT
25	CD160	NKT
25	CD247	NKT

26	PTPRC	NK
26	CD247	NK
26	EOMES	NK
27	GLUL	HCC
28	CD79A	DoubleCell
28	HSPA5	DoubleCell
29	KRT7	HCC
29	GLUL	HCC
29	ARG1	HCC
29	HSPA5	HCC
29	BSG	HCC
29	CDKN2A	HCC
30	KRT19	HCC
30	EPCAM	HCC
30	GLUL	HCC
30	KRT7	HCC
30	BSG	HCC
30	HSPA5	HCC
30	MUC1	HCC
31	ENG	En
32	PTPRC	NKT
32	CD3D	NKT
32	CD3E	NKT
32	CD3G	NKT
32	CD247	NKT
32	TRAV24	NKT
33	GPC3	HCC
33	BSG	HCC
33	CDKN2A	HCC
33	AFP	HCC
33	BIRC5	HCC
34	PTPRC	Neutrophils
34	FCGR3A	Neutrophils
35	PTPRC	ILC
35	NCR1	ILC
35	ID2	ILC
35	RUNX3	ILC
36	HSPA5	HCC
37	GLUL	HCC
37	CHI3L1	HCC
37	GPC3	HCC
38	PTPRC	DoubleCell
38	MS4A1	DoubleCell
38	S100A4	DoubleCell
38	IL7R	DoubleCell
39	MUC1	HCC

40	VCAM1	En
41	PTPRC	MDDC
41	CD1A	MDDC
41	CD1C	MDDC
42	HSPA5	HCC
42	GLUL	HCC
43	FCGR3A	Neutrophils
43	FCGR3B	Neutrophils
44	PTPRC	pDC
44	CLEC4C	pDC
44	IL3RA	pDC
