# Supplementary material for: Single-cell RNA sequencing revealed potential targets for immunotherapy studies in hepatocellular carcinoma
Source: Sci Rep. 2023 Nov 1;13:18799. doi: 10.1038/s41598-023-46132-w (PMC10620237; doi:10.1038/s41598-023-46132-w)
Supplement: Supplementary file 7 — Supplementary Table S4. [file 41598_2023_46132_MOESM7_ESM.pdf]

Supplementary Table 4. HCC cell stemness scores

Subclusters	Cell stemness scores (mean±STD)
HCC_FYB	0.46±0.13
HCC_HP	0.33±0.05
HCC_NTS	0.31±0.07
HCC_HRG	0.31±0.05
HCC_GZMA	0.39±0.07
HCC_GPX2	0.35±0.11
HCC_LTB	0.43±0.11
